# Persister cells formation and expression of type II Toxin-Antitoxin system genes in *Brucella melitensis *(16M)* and Brucella abortus *(B19)

**DOI:** 10.30699/ijp.2020.118902.2294

**Published:** 2020-02-19

**Authors:** Fatemeh Amraei, Negar Narimisa, Behrooz Sadeghi kalani, Vahid Lohrasbi, Faramarz Masjedian Jazi

**Affiliations:** 1 *Microbial Biotechnology Research Center, Iran University of Medical Science, Tehran, Iran*; 2 *Department of Microbiology, School of Medicine, Iran University of Medical Sciences, Tehran, Iran*; 3 *Clinical Microbiology Research Center, Ilam University of Medical Sciences, Ilam, Iran*

**Keywords:** Brucella melitensis, Brucella abortus, Persister cell, TA systems, Real-time PCR

## Abstract

**Background & Objective:**

Persister cells are defined as a subpopulation of bacteria that are capable of reducing their metabolism and switching to dormancy in stress conditions. Persister cells formation has been attributed to numerous mechanisms, including stringent response and Toxin-Antitoxin (TA) systems. This study aimed to investigate the hypothetical role of TA systems in persister cells formation of *Brucella* strains by evaluating toxins of type II TA systems (*RelE*, *Fic*, *Brn* *T*, cogT) expression.

**Methods:**

*Brucella* strains treated with a lethal dose of gentamicin and ampicillin and to determine the number of surviving cells, bacterial colonies were counted at different time intervals. The role of TA systems in persister cell formation was then determined by toxin expression levels using qRT- PCR method.

**Results:**

Our results showed the viability of persister cells after 7 h. The results of relative qRT- PCR showed higher levels of toxin gene expression due to stress conditions, suggesting the possible role of TA systems in persister cells formation and antibiotics tolerance.

**Conclusion:**

The results of this study showed that considering the importance of persistence and the tolerance to antibiotics, further studies on persister cells formation and related genes such as the TA system genes in *Brucella* strains might help us to identify the precise mechanisms leading to persister cells formation.

## Introduction

Brucellosis is a zoonotic disease that causes abortion, genital infections and fetal death in animals. In humans, this highly diverse illness initially presents as fever, malaise, and myalgia and may later develop into a chronic illness affecting various organs and tissues such as the spleen, brain, joints, heart, liver and bone marrow (1-3) .Brucellosis is an endemic disease in the Middle East, the Mediterranean countries and East Asia and Latin America. World health organization (WHO) has reported every year 500,000 new cases of brucellosis diagnosed in above regions. The causative agents of brucellosis are facultative intracellular, coccobacilli, aerobic and gram-negative bacteria belonging to the genus *Brucella* (4,5).

Based on the differences in host specificity and biochemical analysis, ten species are identified in the genus *Brucella* including *B*.* melitensis*, *B*.* abortus*, *B*. *ovis*, *B*. *suis*, *B*. *canis*, *B*. *neotomae*, *B*. *inopinata,* *B*. *ceti*, *B*. *microti*, and *B*. *pinnipedialis* (6). However, the most prevalent pathogenic species in humans and animals are *Brucella melitensis* and *Brucella abortus* (7). Brucellosis disease appears in the forms of the acute, sub-acute or chronic (8). Treatment of chronic infections is complicated by both bacterial persistence and resistance, two distinct phenomena occurring through unrelated mechanisms. Resistance is caused by genetic variations, which result in the alteration of antibiotic targets to reduce antibiotic binding and efficacy. As a result, resistant bacteria are able to survive and even grow in the presence of antibiotics (9). When the antibiotic concentration exceeds a certain threshold, only so called "persister" cells can survive (10). 

These cells were primarily detected in 1944 by Joseph Bigger who noticed the survival of a subpopulation of *Staphylococcus* *aureus* which were being exposed to a lethal dose of penicillin. Persister cells have a significant role in the progression of chronic infections such as tuberculosis, cystic fibrosis, and candidiasis (11,12)

Although there are little data about bacterial persistence, recent studies represent toxin-antitoxin (TA) systems have a major role in persister cells formation (13).

TA systems are abundant small modules in the genomes of bacteria and archaea, generally composed of two elements: a stable toxin that targets an essential cellular process and a labile antitoxin that inhibits the toxins deleterious activity. Several distinct families of TA systems have been identified on bacterial chromosomes that regulate stress survival and persister cells formation. 

TA systems are currently divided into six different classes (I-VI), in which the type II of these systems has mostly been studied. In the type II TA system, two protein genes (toxin and antitoxin) are co-expressed (14, 15). In normal conditions, antitoxin inhibits the activity of the stable toxin by forming a tight protein-protein complex (16). In the stress condition, the labile antitoxin is selectively degraded by cellular proteases such as Lon and clpXP. Destruction of antitoxins makes toxin to target the essential bacterial processes (protein translation, and cell-wall synthesis, DNA replication) and triggers various functions such as switching of bacteria into dormant, stress management, bacterial persistence (17,18).

Therefore, this research aimed to study persister cells formation and expression levels of type II TA system toxin genes in *Brucella* spp, treated with gentamicin and ampicillin.

## Materials and Methods

**Bacterial Strains and DNA Preparation**

In this study, two standard strains of* Brucella* spp, including *Brucella melitensis* (16M) and *Brucella* *abortus *(B19) were used from the reference laboratory and the school of medicine of Iran University of Medical Sciences, Iran. Strains were cultured on Brucella agar (Merck, Darmstadt, Germany) and incubated at 37°C with 5% CO_2_ for 48 h. Genomic DNA extraction was performed using Gene All Kit (South Korea) according to the manufacturer’s instructions. The DNA purity and concentration were measured using a NanoDrop™ spectrophotometer (Thermo Fisher Scientific, Waltham, MA, USA). The absorbance ratio at 260 nm and 280 nm (A260/280) was used to assess the purity of DNA and the ratio of ~1.8 was considered as purity for extracted DNA. DNA was stored at -20°C until the Polymerase chain reaction (PCR) was carried out (4, 19).

**Identification of TA Systems and Primer Design**

Complete Genome sequences of *B. melitensis *(16M) and *B. abortus *(B19) were obtained from NCBI GenBank and TADB database (http://bioinfo-mml.sjtu.edu.cn/TADB/) to identify type II TA systems. Moreover, NCBI BLAST was used to find homologous of type II TA systems. Speciﬁc primers were designed by using OLIGO software V. 7.56 (Table 1).

**Table 1:** Primers used for both PCR and qRT-PCR studies

Primer Name	Primer sequence (5′→3′)	Ampliconsize (bp)	Annealing temperature	Reference
***RelE***	F: TGCAATTGAGCGAAGATTTG	208	57°C	In this study
	R: TCATCCGGTGGTAAGATTGG			
***Fic***	F: CATCAGGAACAACTGCGCTT	111	59°C	In this study
	R: ATCGGGATTGCCATAGGCTT			
***Brn T***	F:ACAGACCAACATTGCCAAGC	106	59°C	In this study
	R: GATTGCCATCAGGCGATCTG			
***CogT***	F: ATCTGGAAGCCGTCATGGAA	122	59°C	In this study
	R:GGCCATGACAATGCTGGAAT			
***16S rRNA***	F: CAGATTACGCAAGCAGCCTT	134	58°C	In this study
	R: TCCTCGACGCTTAGTGTCTC			

**PCR Analysis of TA System Genes**

PCR assay was used to detect the presence of the type II TA system genes (*RelE*, *Fic*, *Brn T*, *Cog* *T*) in *Brucella* spp.

PCR amplifications was performed in a final volume of 25 µL. Each PCR reaction mixture contained 8 μL Master mix 1X (Ampliqon Co, Denmark), 1 μL template DNA (0.5 μg), 1 µL of each forward and reverse primers and sterile distilled water up to 25 μL.

PCR amplification was performed in a DNA thermal cycler (PeqLab, Germany). PCR program consisted of an initial denaturation step at 95°C for 4 min; 35× 95°C for 45 s, annealing at 60°C for 45 s, and extension at 72°C for 30 s, with a final extension step at 72°C for 3 min. PCR products were purified and analyzed on 1.5 % (w/v) agarose gel in TBE buffer at 85 volt and 25 mA, for 40 min using the 50 bp ladder (Pishgam, Iran) as the molecular weight standard (5).

**Determination of Minimum Inhibitory Concentration (MIC)**

The microdilution method was used to determine the MIC of *Brucella* spp, according to the National Committee for Clinical Laboratory Standards (NCCLS). The study was done in accordance with good clinical practice under a biocontainment level 3 biosafety cabinet (20). To determine the MICs of gentamicin and ampicillin (Sigma Aldrich), 100 μL of 0.5 McFarland overnight bacterial culture (dilution of 1:100 in MHB) and different concentrations of antibiotic solution were added to the 96-well microtiter plates and incubated at 37°C with 5% CO_2_ for 48 h. 

The MIC was considered as the lowest concentration of antibiotic that prevented the visible bacterial growth after 48 h of incubation. MIC values for the studied antibiotics were independently determined three times (21).

**Persister Cell Formation Assay**

To measure the number of persister cells, *B. melitensis *(16M) and *B. abortus* (B19) was cultured overnight and diluted to the proportion of 1:100 in tryptic soy broth. Bacterial culture was incubated at 37°C with 5% CO2 on a shaker at 200 rpm until the absorbance at 600 nm reached 0.25 (logarithmic phase). Then, was added to the culture at a concentration of gentamicin 40 µg/mL (60x MIC) and 100 µg/mL (50x MIC) of ampicillin. Then, 1 mL of culture was washed twice by 0.85% sterile saline solution to remove the antibiotic after 1, 3, 5, 7, and 18 h. Serial dilution of bacterial culture was prepared and each dilution was poured onto Brucella agar. Plates were then incubated at 37°C with 5% CO_2_ for 48 h. Finally, colonies were counted in order to detect the presence of the bacterium on agar plate.

**Quantitative Real-Time PCR** **(qRT-PCR)**

Total RNA of *B. melitensis* (16M) and *B. abortus* (B19) was extracted using high pure RNA isolation kit (Roche kit, Germany) 7h after of treatment with antibiotics gentamicin and ampicillin. Total RNA of untreated *B. melitensis* (16M) and *B. abortus* (B19) was also extracted as control. The quantity and quality of RNA were assessed using the NanoDrop spectrophotometer (Thermo Fisher Scientific, USA) and gel electrophoresis analysis. In order to remove DNA contamination, extracted RNA was treated with DNase1 (Roche, Germany) according to the manufacturer’s protocol. Total RNA was reverse transcribed to cDNA using the cDNA Synthesis Kit (Takara, Japan).

The qRT-PCR (three replicates) assay for each gene was performed on Rotor-Gene thermal cycler according to the following program: one cycle of 95°C for 12 min, 40 cycles of 95˚C for 10 s, and one cycle of 60°C for 45 s. 

A housekeeping gene called 16S rRNA was used as the internal control for normalization of mRNA levels and fold changes and mRNA expression was calculated by the 2^ (-∆∆CT) method (22).

**Statistical Analysis**

Data obtained from the mRNA expression analysis were presented as means ± standard error of three independent assessments. Values obtained for the expression of each individual gene exposed to antibiotics were compared using one-way analysis of variance (ANOVA) test followed by Dunn-Test and Friedman test for multiple comparisons by Prism 8 (GraphPad Software, Inc).

## Results

**TA System Genes** **Detection by PCR**

**Fig. 1:**
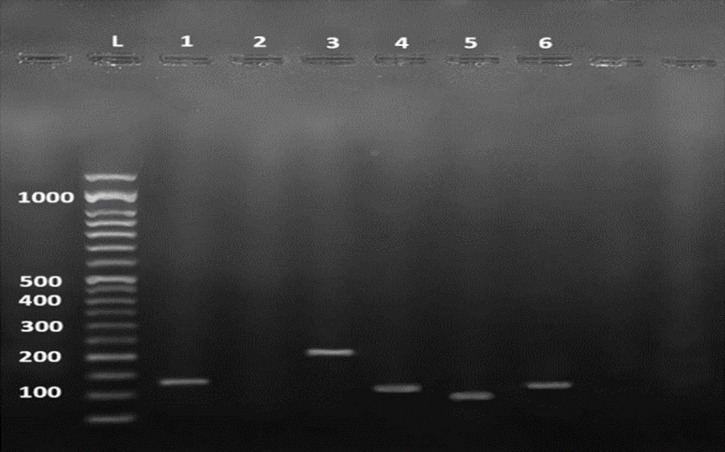
The presence of TA systems detection by PCR in *Brucella*. Lanes 1-6 respectively contains: 50 bp DNA ladder, 1: positive control 16S rRNA gene (134 bp), 2: negative control, 3: *RelE* (208 bp), 4: *Fic* (111bp), 5: *Brn T* (106 bp) ,6: *CogT* (122 bp).

We investigated the existence of type II TA systems in *B. melitensis* (16M) and *B. abortus* (B19). Our results revealed the presence of all of the studied genes coding for type II TA system in this strains (Figure 1).

**Antimicrobial Susceptibility Testing in** ***Brucella*** **spp.**

In this study, we first determined the MIC ranges of gentamicin and ampicillin against *Brucella* spp, using the broth micro dilution assay. The MIC ranges for the selected antibiotics were for *Brucella melitensis* (16M) and *Brucella abortus* (B19) 2 μg/mL and 0.5 μg/mL, respectively.

**Persister Cells Populations in** ***Brucella*** **spp.**

**Fig. 2:**
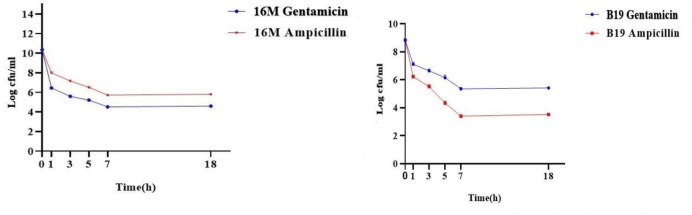
Stabilization test for bacteria through colony count (CFUs). (Fig. 2 A) Experiment for persister cells heritability using gentamicin and ampicillin antibiotics against *Brucella melitensis* (16M) and (Fig. 2 B) *Brucella abortus*

As shown in Figure 2, to evaluate persister cells *Brucella* spp, culture was treated with gentamicin and ampicillin during the exponential phase. Colonies were counted and colony forming unit calculations indicated that after 1 h, the number of viable cells reduced significantly, while after 7h, the number of viable cells remained the same. Survival curves of plateau-phase demonstrated the survival of persister cells.

persister cells of 16M and B19 with lethal concentration of gentamicin and of ampicillin were performed at different times to assess the number of colonies that survived the cells. Error bars indicate standard deviation of three independent replicates (*P*<0.01 by Friedman test).

**Relative Gene Expression**

**Fig. 3:**
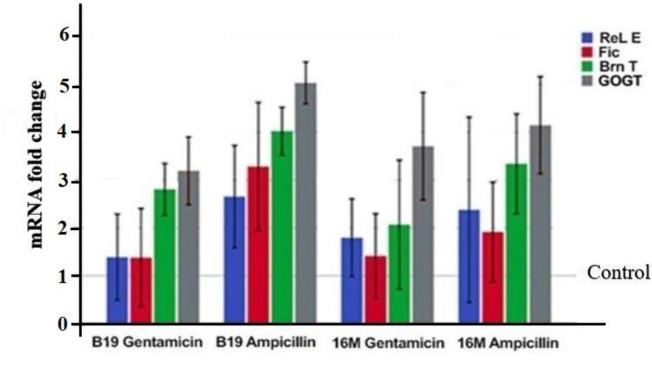
Gene expression analysis by Real-time PCR. Relative expression is normalized for *Brucella melitensis* (16 M) and *Brucella abortus* (B19) with housekeeping 16S rRNA gene (*P* <0.05)

Our qRT-PCR data analysis showed that all the studied type II TA systems (*RelE*, *Fic*, *Brn* *T*, *CogT*) in the selected isolates had a higher expression levels than the control sample (Figure 3).

Furthermore, statistical analysis results showed that levels of TA expression after antibiotics treatment in the four groups (*RelE*, *Fic*, *Brn T*, *CogT*) was statistically significant at 95% confidence levels (*P*<0.05) (Figure 3).

Bars represent the mean ± SD of three independent replicates (*P*<0.05 by Friedman test).

## Discussion

 According to studies, persister cells are responsible for antibiotic tolerance, bacterial survival in biofilms, and development of recurrent infection caused by different bacteria (23,24). In our study, we examined persister cells formation in *Brucella* spp, treated with gentamicin and ampicillin. Considering the association between type II TA system toxins and persistence, after the treatment of *Brucella* spp, with a lethal dose of gentamicin and ampicillin, to determine the effect of TA systems on persister cells formation, RNA of *Brucella* spp, was extracted after 7 h treatment with gentamicin and ampicillin and expression levels of four type II TA system toxins were measured using relative qRT-PCR. 

However, persister cells for *Brucella* spp, were formed after challenge with antibiotics and the results showed that the number of viable cells was altered according to the number of colony count. Furthermore, our results showed that increase in transcription levels of *RelE*, *Fic*, *Brn T*, *CogT* compared to the control strains. Previous studies have indicated the role of TA systems in persister cell formation. TA systems were at first identified in plasmids accounting for post-segregational killing mechanism (25). Thereafter, many of these systems were found on bacterial chromosomes. Chromosomal TA systems affect essential cellular mechanisms by which bacterial cells can survive in stress conditions (26). 

According to studies, six different classes of TA systems have been identified among which type II systems is known to have a role in persister cells formation. In type II TA systems, protein antitoxin has two domains; one domain binds a palindromic sequence of the promoter leading to the repression of type II TA operon, while the second domain binds the cognate toxin leading to its inhibition (17). In the stress condition, unstable antitoxin is degraded by proteases such as ClpXP and ClpAP, hence the free toxin can affect major cellular processes leading to bacterial persistence and dormancy (27). 

Under stress conditions, most type II toxins are able to inhibit translation by different mechanisms. For instance, *vapC* toxin cleaves tRNA^fMet^ by its RNase activity, which can further block protein synthesis. The *Doc* toxin binds the 30S ribosomal subunit leading to the inhibition of translation and growth arrest in bacterial cells (28).

Harris Moyed et al, in 1983 reported that two mutations in *hipA* gene increased persistence in *E.* *coli* for 1000 fold. *HipA* gene in *E. coli* was recognized as the first gene of TA system that plays a role in persistence (29). *HipA* is a toxin of *hipAB* TA system that phosphorylates elongation factor Tu (EF-Tu) by its kinase activity, causing reduction in translation and induction of persistence (30, 31). Vázquez-Laslop *et al.*, demonstrated that the ectopic expression of *RelE* and *MazF* toxins in *E. coli* can lead to bacterial antibiotic tolerance and persistence (32). 

In another study, it was shown that exposure to ciprofloxacin in *E. coli* increased the level of *TisB* toxin and increased the number of persister cells. However, the most direct evidence for a role of TA systems modules in persister cells formation is derived from studies showing that mutations in the *hipBA* TA system of *E. coli* can modulate the frequency of persister cell formation. Similarly, it has been reported that inactivation of the *RelE* genes in *Mycobacterium* *tuberculosis* may influences on the frequency of persister cells formation (33).

Our results showed increase in transcription levels of *RelE*, *Fic*, *Brn* *T*, *CogT* compared to the control strain, suggesting the possible role of TA systems in persistence and antibiotic tolerance. 

Furthermore, our study showed increase in *CogT* gene expression which was the highest expression level among the studied toxins, suggesting that toxin *CogT* may have the greatest effect on the production of persistent cells in *Brucella* spp. PSI-BLAST results showed that *CogT* contains three highly conserved polar groups that could form an active site. These are an arginine, glutamate and serine, the RES domain. RES is found widely distributed in bacteria.

 Since antibiotic tolerance caused by persister cells results in treatment failure and chronic infections, investigating the genes related to persistence such as TA systems help us to recognize the precise mechanisms linked to persistence.

## Conclusion

Chronic brucellosis is a major problem in medical science, as it causes many complications in patients. According to the results, there may be a link between the persistence of this bacterium and the TA system genes, which can be a way of treating the disease. The results of this study indicate that *RelE*, *Fic*, *Brn T*, and *CogT* genes may play a role in the persistence of the *Brucella* bacteria. Also, the *CogT* gene expression has increased in comparison with other genes in two standard strains. But the mechanism of this *CogT* system is still unknown. Although further studies are needed to determine the exact role of these genes, including; extruding these genes, making mutant strains, examining animal models, and cell culture studies.
